# Complete resection of brain metastases – when does it matter?

**DOI:** 10.1007/s11060-025-05193-9

**Published:** 2025-08-22

**Authors:** Tommaso Araceli, Amer Haj, Christian Doenitz, Eva-Maria Stoerr, Katharina Rosengarth, Nils Ole Schmidt, Martin Proescholdt

**Affiliations:** 1 Department of Neurosurgery, University Regensburg Medical Center, Regensburg, Germany; 2 Wilhelm-Sander Neuro-Oncology Unit, University Regensburg Medical Center, Regensburg, Germany

**Keywords:** Gross total resection, Brain metastasis, Survival analysis, Extent of resection

## Abstract

**Purpose:**

The value of gross total resection (GTR) in patients with brain metastases (BM) is controversial. Therefore, we analyzed the circumstances under which GTR is crucial for optimal outcome in a large population of patients with BM treated with surgical resection at our institution.

**Methods:**

The analysis included 539 patients. The extent of resection was rated as complete if no residual contrast-enhancing tumor was detectable on the early postoperative magnet-resonance image (MRI); the tumor size was determined by measuring the volume of the contrast-enhancing areas on the presurgical MRI. Outcome included overall survival (OS) and progression-free survival (PFS).

**Results:**

GTR was achieved in most patients (82.8%) but was not associated with longer OS and PFS in the entire population (HR: 0.88; *p* = 0.162 and HR: 0.84; *p* = 0.319). However, a significant survival benefit of GTR was observed in patients with solitary BM (HR: 0.39; *p* = 0.0006). Age younger than 65 years (HR: 0.75; *p* = 0.047), controlled disease status (HR: 0.68; *p* = 0.033), focal radiotherapy (HR: 0.64, *p* = 0.044), postsurgical systemic treatment (HR: 0.67; *p* = 0.038), and no target therapy (HR: 0.75, *p* = 0.039) were also associated with significant benefit of GTR. Multivariate interaction analysis showed that solitary BM and controlled disease status significantly influenced the impact of GTR in our patient population (*p* = 0.0001).

**Conclusion:**

Achieving GTR is highly relevant in patients with solitary BM status, controlled systemic disease, specific postsurgical systemic treatment options, postsurgical focal radiation strategies, and in the population younger than 65 years of age.

**Supplementary Information:**

The online version contains supplementary material available at 10.1007/s11060-025-05193-9.

## Introduction

Brain metastases (BM) develop in 20–40% of patients with cancer, making them the most frequent type of brain tumor in adulthood [1]. As the incidence of BM increases, so does the need for a consistent, accurate understanding of the associated epidemiologic factors [2]. The survival prognosis is poor after diagnosis of BM [3] and factors such as the number of BM, the detection of systemic metastases, the general clinical condition, and the psycho-oncological burden greatly influence the outcome [4, 5]. Surgical treatment plays a central role in the management of BM [6, 7, 8, 9, 10, 11, 12]. The surgical resection of BM reduces mass effects and intracranial pressure, prolongs overall survival (OS), decreases symptom burden as well as focal neurological deficits due to the decompression of eloquent areas of the brain, normalizes the metabolic microenvironment [13] enhances the potential quality of life improving postsurgical Karnofsky Score (KPS) and the Recursive Partitioning score (RPA) [14] and increases the probability for the patient to receive adjuvant local and systemic treatment [15]. Additionally, surgery of BM may help optimize seizure control, by reducing cortical irritation [16]. However, the importance of gross total resection (GTR) is still a topic of controversial debate, given that most patients with BM die from systemic disease progression [17]. This study analyzes the conditions under which GTR improves outcomes in a large population of patients with BM treated with surgical resection at our clinic.

## Materials and methods

### Study design and ethical approval

The clinical data of patients with BM who had undergone surgical resection of one or more BM lesions at our center between January 2016 and January 2024 were collected using a prospectively collected registry database. Patients with missing pre- or postoperative magnet-resonance image (MRI), unknown extent of resection (EOR), previous treatment of BMs, or missing follow-up data were excluded. All cases were discussed by our interdisciplinary neurooncological board with representatives from all disciplines involved in the complex therapy of BM. The decision to operate was made considering several factors: the patient’s overall performance score, and therefore implicitly the degree of their comorbidities, and the presence of a symptomatic and space-occupying mass with significant perilesional edema and/or blockage of the cerebrospinal fluid pathways. Ethical approval from the Regensburg University Institutional Ethics Review Board was obtained (protocol no. 20-1799-101) in accordance with German ethical and regulatory standards and the Declaration of Helsinki (7th revision, 2013). The data protection concept at the Brain Tumor Center Regensburg, established according to the European General Data Protection Regulation and relevant national legislation, was strictly followed.

### Data collection and definitions

The following variables were collected from the electronic patient files of the SAP^®^ software (SAP^®^ Deutschland SE & Co.KG, Walldorf, Germany) and the radiological, oncological, medical, and tumor board reports: age, gender, histology of the primary tumor, BM timing, location of BM, BM status (solitary = one single BM without systemic metastases, singular = one singular BM and at least one systemic metastasis, and multiple = more than one BM), systemic disease control status, pre-and postoperative KPS, RPA considering age, KPS, primary tumor control and presence of extracranial metastases, pre- and postoperative volume of the resected BM, use of corticosteroids, pre- and postsurgical symptoms such as hemisymptoms (HS) = sensory-motor deficits on one side, aphasia and visual field impairments (VF), and postsurgical treatment. With regard to systemic treatment, three subgroups were considered: chemotherapy (CT), immunotherapy (ICI), and target therapy (TT). The following radiation therapies were considered: whole brain radiation therapy (WBRT), focal radiation therapy (FRT), and stereotactic radiosurgery (SRS). All patients were followed up from the time of BM diagnosis until death or last follow-up. We considered OS and intracranial progression-free survival (PFS) as outcome measure, calculated from BM diagnosis to the date of death or last follow-up or the date of intracranial recurrence. Using RECIST (version 1.1) and iRANO criteria as radiologic tumor response, the systemic disease was considered controlled when therapeutic intervention led to stable disease without proof of progression [18]. Complete resection was defined as the absence of any detectable residual tumor on an early postoperative MRI with T1-weighted Gadolinium-enhanced sequences (within 48 h after surgery) as well as by volumetric analysis obtained with the software ITK-SNAP [19]comparing pre- and postoperative MRI. Otherwise, the resection was defined as subtotal (STR). Three-dimensional visualization was realized using Brainlab software (Munich, Germany) (Supplementary Fig. 1). We considered the tumor to be small when its volume was less than the median of all tumor volumes measured in our cohort, which equals 16.19 ml. The eloquent areas were defined according to the established classification of eloquent cerebral structures as brain areas with readily identifiable neurological functions, the injury of which results in disability [20].

### Definition of endpoints

The primary endpoint of the study was OS, which was used to assess the prognostic impact of GTR in patients undergoing surgical treatment of BM. Secondary endpoints were OS and PFS in clinically relevant subgroups.

### Statistical analysis

The statistical analysis was performed using Stata/IC (version 16.1, Stata Corp. College Station, USA). Descriptive statistics included continuous variables, such as medians and ranges, and categorical values, such as counts and percentages. Univariate analysis was performed by calculating log-rank tests. Survival analysis was done using the Kaplan–Meier method. Multivariate interaction analysis was performed to identify independent prognostic factors using a Cox proportional hazards model. Results with *p* < 0.05 were considered statistically significant.

## Results

### Baseline patient characteristics

Baseline was defined as time of the histologic diagnosis of BM after neurosurgical resection and the first follow-up. Median follow-up time was 15.55 months. In the entire cohort, 539 patients (247 women, 292 men) with a median age of 62.8 years (range: 23.4–86.2 years) were identified. The median preoperative KPS was 80 (range: 30–100); 254 patients (47.1%) showed control of the systemic disease and 285 (52.9%) did not; 349 (64.8%) received at least one type of postsurgical systemic treatment, and 190 (35.2%) did not; 34.9% of patients received CT, 14.8% ICI, and 18.0% TT. WBRT was used in 42.1% of cases, FRT in 27.5%, and SRS in 5.2% of cases; 389 (72.2%) patients received preoperative steroids and 497 (92.2%) postoperative steroids. With regard to BM status, 245 patients (45.5%) presented with multiple BMs, 198 (36.7%) with singular BM, and 96 (17.8%) with solitary BM; 344 patients (63.8%) were treated for metachronous BM, and 195 patients (36.2%) for synchronous BM. The most frequent primary tumor was lung cancer (*n* = 201, 37.3%), followed by melanoma (*n* = 79, 14.6%), and breast cancer (*n* = 67, 12.5%). Baseline characteristics are summarized in Table 1. The overall impact of adjuvant therapy on survival is presented in Supplementary Table 1.

**Table 1:** Patient characteristics at baseline. *Values are given as number of patients (%) or median (range)*

Parameter	Value
Total population	539
Gender (f/m)	247/292 (45.8%/54.2%)
Age (median)	62.8 (range: 23.4–86.2)
Preoperative KPS (median)	80 (range: 30–100)
Systemic control (yes/no)	254/285(47.1%/52.9%)
Postsurgical systemic treatment (yes/no) Chemotherapy/ immunotherapy/ target therapy	349 (64.8%)/ 190 (35.2%) 188(34.9%)/ 80 (14.8%) / 97 (18.0%)
Radiotherapy (whole brain radiation therapy/ focal radiation therapy/ stereotactic radiosurgery)	227(42.1%)/ 148(27.5%)/ 28(5.2%)
Metastasis timing (synchronous/metachronous)	195/344 (36.2%/63.8%)
Metastasis status (solitary/singular/multiple)	96 (17.8%)/ 198(36.7%)/ 245(45.5%)
Primary tumor (lung/melanoma/breast/other)	201 (37.3%)/ 79(14.6%)/ 67(12.5%)/ 192(35.6%)
Corticosteroids use preoperative (yes/no/n.a.)	389 (72.2%)/ 51(9.4%)/ 99(18.4%)
Corticosteroids use postoperative (yes/no/n.a.)	497 (92.2%)/ 20(3.7%)/ 22(4.1%)

### Achievement of GTR

GTR was achieved in 446 patients (82.8%), STR in 93 patients (17.2%). GTR was achieved in 288 patients (80.2%) with large tumors and in 156 patients (88.6%) with small tumors, in 326 patients (84.7%) in non-eloquent areas and in 118 patients (78.7%) in eloquent areas. Univariate analysis showed that GTR was significantly more often achieved in the case of small tumors (*p* = 0.015) and in non-eloquent areas (*p* = 0.37). No statistically significant association between GTR and RPA classes (*p* = 0.168) was found.

### Relation between GTR and neurological worsening

In the entire population, 46 patients (8.5%) experienced neurological worsening. In the group of patients with GTR, 34 (7.7%) had neurological worsening, as did 12 (12.7%) in the group with STR. Patients with GTR had a slightly lower rate of neurological worsening than patients without GTR, but, according to the chi-squared test, the difference in neurological worsening was not statistically significant between the two groups (*p* = 0.115). These data are summarized in Supplementary Table 2.

### Relation between GTR and functional outcomes

Patients with STR or GTR were compared across a range of postoperative functional outcomes. HS did not differ significantly between groups (*p* = 0.126), although stable outcomes were more frequent in the GTR group (38.2%) than in the STR group (20.0%), and worsened outcomes occurred more often in the STR group (20.0%) than in the GTR group (6.6%). Even VF were similar between the two groups (*p* = 0.724), with both showing high rates of stable status (STR 60.0% and GTR 62.3%). In contrast, aphasia analysis revealed a statistically significant difference (*p* = 0.039), as the GTR group more frequently showed aphasia resolution (GTR 38.8% vs. STR 5.9%) and less worsening (4.1% vs. 17.7%), suggesting more favorable language outcomes after complete resection. Overall, postoperative KPS scores were significantly higher than preoperative KPS scores (mean Δ=+3.93, *p* < 0.0001), reflecting general functional improvement after surgery. In a subgroup analysis, the KPS in the STR group improved from 77.6 to 81.2 (mean Δ=+3.63, *p* = 0.016), in contrast to 79.8 to 83.9 (mean Δ=+4.08, *p* < 0.0001) in the GTR group. The two groups showed no significant differences between groups in preoperative (*p* = 0.213) or postoperative KPS scores (*p* = 0.179), although scores in the GTR group were consistently higher. The KPS score gain (postsurgical − presurgical) confirmed this trend, with the GTR group showing a slightly larger improvement (mean Δ=+3.92) than the STR group (mean Δ=+3.19). Similarly, KPS categorical outcomes showed no significant differences (*p* = 0.603), with comparable improvement rates (STR 42.7% vs. GTR 48.7%) and worsening (STR 9.8% vs. GTR 9.0%) in the two groups. These data are summarized in Supplementary Table 2.

### Relation between GTR and use of corticosteroids

In the STR group, 63 (80.8%) patients had received steroid treatment preoperatively, while 15 (19.2%) had not. In the GTR group, 321 (89.9%) patients had received preoperative steroid treatment preoperatively and 36 (10.1%) had not. Postoperatively, 81 (92.0%) patients in the STR group received postoperative steroids and 7 (8.0%) did not. In the GTR group, 411 (96.9%) patients received steroids postoperatively and 13 (3.1%) did not. The use of preoperative (p=0.023) and postoperative (p=0.031) steroids was more common in patients with GTR. The stratification by corticosteroid use revealed no significant association between GTR and OS in either the preoperative group (HR: 0.87, *p* = 0.333, with steroids; HR: 0.59, *p* = 0.134, without steroids) or in the postoperative group (HR: 0.88, *p* = 0.296, with steroids; HR: 0.66, *p* = 0.468, without steroids) groups. These data are summarized in Supplementary Table 3.

### Impact of GTR on OS and PFS

**Fig. 1:**
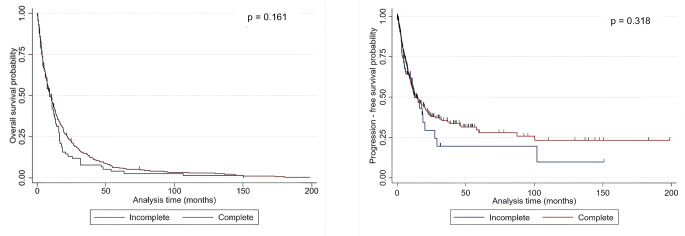
Kaplan-Meier plot depicting the influence of the EOR on OS and PFS in the entire population

The mean OS for the patients with GTR was 18.4 ± 28.5 months, whereas the mean OS for the patients with STR was 13.1 ± 20.9 months. The mean PFS for the patients with GTR was 13.5 ± 24.7 months, in contrast to 9.9 ± 19.3 months for the patients with STR. The log-rank test showed no significant difference in terms of OS (*p* = 0.161) and PFS (*p* = 0.318) in the entire population (Fig. 1). Consistent with this, univariable Cox regression analysis showed no significant impact of GTR on OS (HR: 0.88, 95% CI: 0.74–1.05, *p* = 0.162) and PFS (HR: 0.84, 95% CI: 0.59–1.19, *p* = 0.319) (Supplementary Tables 4,6).

### Role of GTR in specific circumstances

#### Patients younger than 65 years

Patients younger than 65 years with STR had a mean OS of 13.2 ± 18.1 months compared to 19.2 ± 28.7 months in patients with GTR; patients older than 65 years with STR had a mean OS of 13.7 ± 24.9 months compared to 16.6 ± 28.2 months in patients with GTR.

**Fig. 2:**
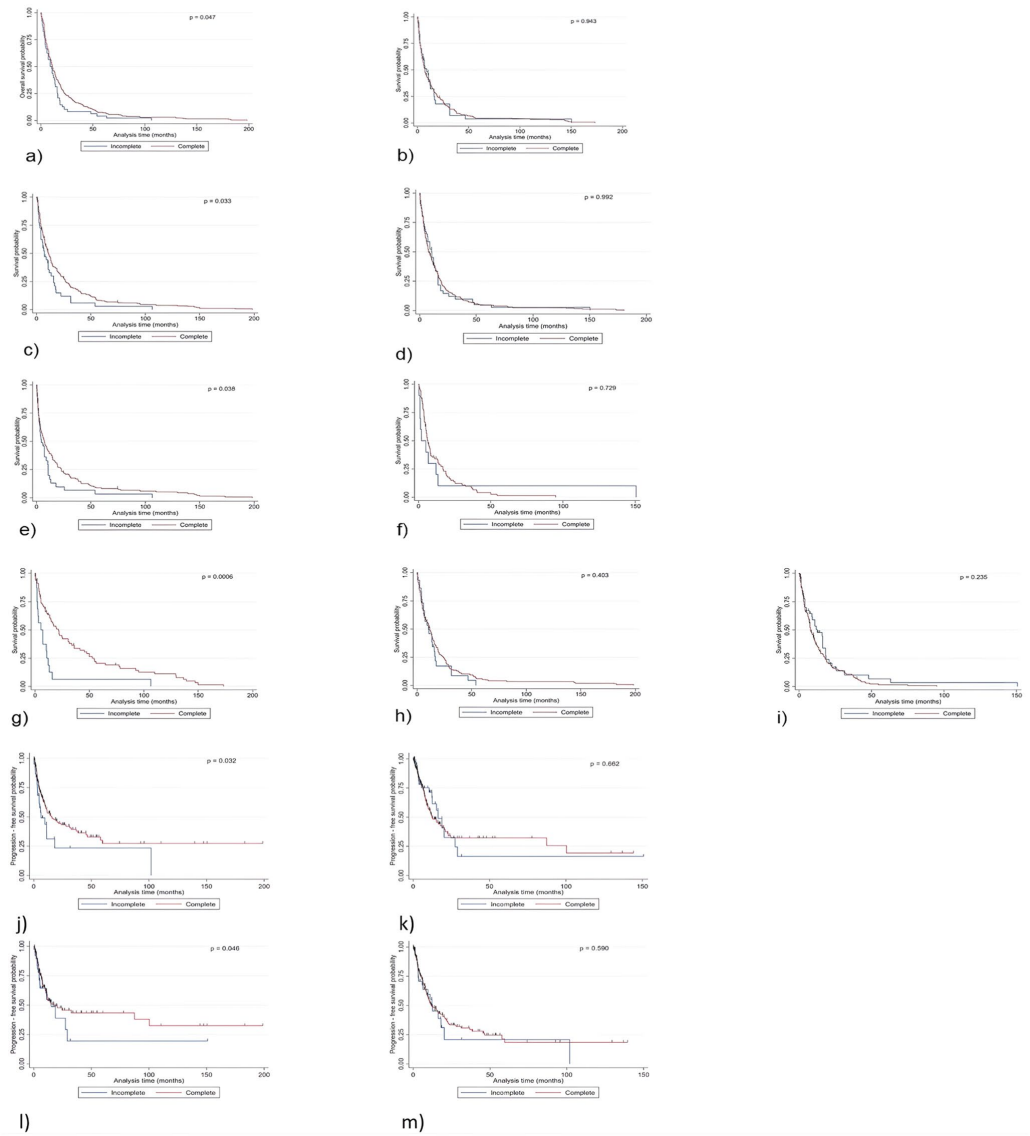
Kaplan-Meier plot depicting that (**a**) GTR significantly influenced OS in patients younger than 65 years; (**b**) GTR was not significantly correlated with OS in patients older than 65 years; (**c**) GTR significantly influenced OS in patients with systemic disease control, in contrast to (**d**) patients without systemic disease control; (**e**) GTR significantly influenced OS in patients with and (**f**) without postoperative systemic treatment; (**g**) GTR significantly correlated with OS in patients with solitary BM; (**h**) GTR was not significantly correlated with OS in patients with a singular BM and (**i**) multiple BM; (**j**) GTR was significantly correlated with PFS in patients with systemic disease control, in contrast to (**k**) patients without systemic disease control; (**l**) GTR significantly influenced PFS in patients with postsurgical WBRT, in contrast to (**m**) patients without postsurgical WBRT

In the Cox proportional hazards model, the covariate “younger than 65 years” was associated with a decreased hazard ratio for death (HR: 0.75, 95% CI: 0.55–1.02, *p* = 0.047). In contrast, in patients older than 65 years, GTR did not influence OS (*p* = 0.943) (Fig. 2a, b and Supplementary Table 4). However, age showed no significant correlation with GTR in terms of PFS (Supplementary Table 6).

#### Controlled disease status

Without systemic disease control, the mean OS in patients with STR was 14.0 ± 23.3 months compared to 15.3 ± 24.9 months in patients with GTR. With systemic disease control, the mean OS for patients with STR was 12.1 ± 18.1 months and 21.9 ± 28.2 months in patients with GTR. Mean PFS for patients with systemic disease control in the STR group was 8.3 ± 16.4 months, in contrast to 16.6 ± 29.4 months in the GTR group. A decreased hazard ratio for death was attested in the covariate “systemic disease control” when associated with GTR (HR: 0.68, 95% CI: 0.48–0.97, *p* = 0.033). However, without disease control, GTR did not improve OS (*p* = 0.992). In patients with systemic disease control, GTR was associated with significantly improved PFS (HR: 0.59, 95% CI: 0.37–0.96, *p* = 0.032). This association was not observed in patients without systemic control (*p* = 0.662). These results are illustrated in the graphics (Fig. 2c, d,j, k and Supplementary Tables 4, 6).

#### Postsurgical systemic treatment

Our data demonstrate that the mean OS for patients who received postsurgical systemic therapy after STR was 9.8 ± 19.2 and 20.7 ± 35.5 months after GTR. In contrast, patients without postsurgical treatment lived an average of 19.4 ± 46.3 months after GTR and 11.4 ± 14.5 months after STR.

In the Cox proportional hazards model, the covariate “postsurgical systemic treatment” showed a decreased hazard ratio for death when associated with GTR (HR: 0.67, 95% CI: 0.46–0.98, *p* = 0.038). In contrast, in patients without postsurgical systemic therapy, GTR did not influence OS (*p* = 0.729) (Fig. 2e, f and Supplementary Table 4).

#### Stratification by systemic treatment modalities

In the STR group, 39 patients (42.9%) received CT, and 52 (57.1%) did not. In the GTR group, 149 patients (33.6%) received CT, while 295 (66.4%) did not. The use of CT was more common in patients with STR, but the difference was not statistically significant (*p* = 0.091). ICI was given to 16 patients (17.6%) with STR and to 64 patients (14.4%) with GTR. The majority of patients in both groups did not receive ICI (STR 82.4% and GTR 85.6%) without any significant difference (*p* = 0.440). TT was used in 23 patients (25.3%) with STR and in 74 patients (16.7%) with GTR with a slight trend toward more TT in the STR group (*p* = 0.052).

Stratified Cox proportional hazards models were performed across various treatment subgroups. In patients who did not receive CT, GTR was not significantly associated with OS (HR = 0.88, *p* = 0.400). However, patients who received CT, showed a trend toward improved survival with GTR (HR: 0.70, 95% CI: 0.48–1.03, *p* = 0.070). A similar trend was observed in patients who received ICI: GTR was associated with a lower risk of death (HR = 0.64, 95% CI: 0.35–1.20, *p* = 0.167), although the results did not reach statistical significance. No significant effect of GTR was observed in the ICI-naïve group (HR: 0.85, *p* = 0.225). Notably, a significant survival benefit from GTR was observed in patients who did not receive TT: here GTR was associated with a lower risk of death (HR = 0.75, 95% CI: 0.57–0.99, *p* = 0.039). However, in the subgroup receiving TT, GTR showed no survival benefit (HR = 1.05, *p* = 0.845). All interaction analyses between EOR and postoperative systemic treatments in terms of PFS showed beneficial trends if GTR was achieved, although these benefits were not statistically significant (Supplementary Table 6).

#### Postsurgical radiotherapy

With regard to stratification by radiotherapy modalities, 38 (41.8%) patients in the STR group received WBRT, while 53 (58.2%) did not. In the GTR group, 187 patients (42.1%) received WBRT and 257 (57.9%) did not, indicating a similar distribution of WBRT use (*p* = 0.95). Use of SRS was low across the cohort (*n* = 28) without any significant distribution difference (*p* = 0.634). With regard to FRT, 33 patients (36.3%) in the STR group received FRT compared to 58 (63.7%) who did not. In the GTR group, 114 patients (25.7%) received FRT and 330 (74.3%) did not. FRT was performed more frequently to patients with STR (*p* = 0.039). GTR did not significantly impact the survival of patients treated with WBRT (HR: 0.91, *p* = 0.611), nor that of patients who did not (HR: 0.79, *p* = 0.151). In the subgroup receiving FRT, GTR was significantly associated with improved survival (HR: 0.64, 95% CI: 0.41–0.99, *p* = 0.044), but this effect was dissipated in patients who did not receive FRT (HR:0.89, *p* = 0.403). GTR was not significantly associated with OS in patients who did not undergo SRS (HR:0.83, *p* = 0.145), nor in patients who did (HR:1.17, *p* = 0.780). Regarding PFS, however, the interaction between EOR and WBRT was statistically significant (*p* = 0.046), suggesting a synergistic benefit with GTR (Fig. 2l, m and Supplementary Table 6).

#### Metastasis status

Another specific condition to define the role of GTR is the detection of systemic metastases. In our study, the mean OS in patients with solitary BM with STR was 12.6 ± 25.4 months and 34.2 ± 42.5 months after GTR. In patients with singular BM, the mean OS was 12.6 ± 12.3 months after STR and 19.1 ± 30.8 months after GTR. In patients with multiple metastases, the mean OS was even lower, reaching 14.4 ± 24.8 months after STR and 11.7 ± 13.4 months after GTR.

In the Cox proportional hazards model, solitary metastases were associated with a decreased hazard ratio for death in patients with GTR (HR: 0.39, 95% CI: 0.22–0.68, *p* = 0.0006). In contrast, in patients with singular and multiple BM, GTR did not influence OS (*p* = 0.403 and *p* = 0.235). These results are illustrated in Fig. 2g-i. No additional statistically significant associations with PFS were observed (Supplementary Table 6).

**Fig. 3:**
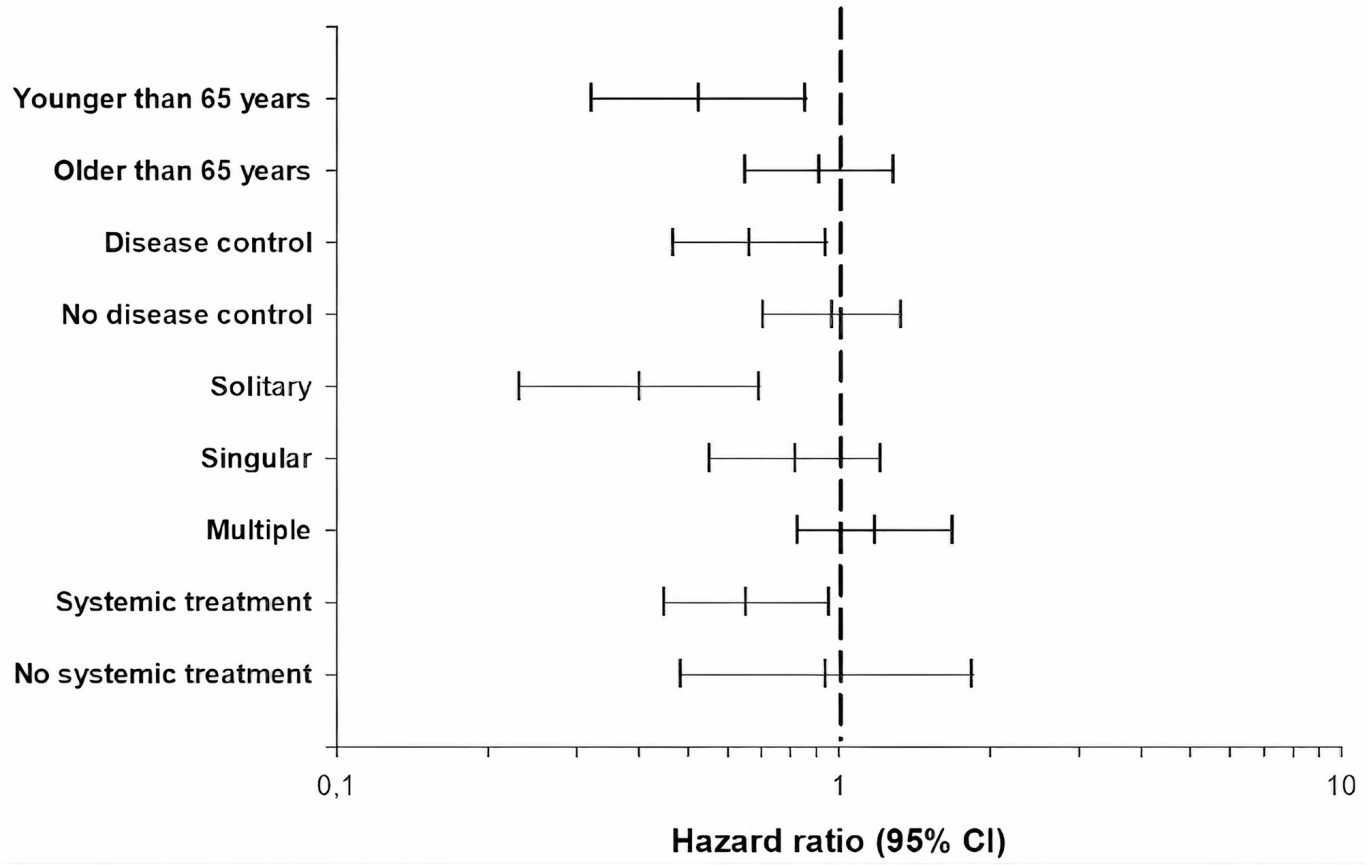
Forrest plot illustrating the impact of GTR under specific conditions. Patients under 65 years of age, with solitary metastases, controlled disease status, and patient who received postsurgical systemic treatment show a sharp significant reduction in HRs of death

The impact of GTR under specific conditions with the corresponding hazard ratio (HR) values were evaluated in a multivariate model and are summarized in Table 2; Fig. 3. A marked reduction in HRs of death is found in patients under 65 years of age (HR: 0.53, 95% CI: 0.32–0.86, *p* = 0.011), with solitary metastases (HR: 0.40, 95% CI: 0.23–0.69, *p* = 0.0001), controlled disease status (HR: 0.66, 95% CI: 0.47–0.94, *p* = 0.023), and with postsurgical systemic treatment (HR: 0.65, 95% CI: 0.45–0.95, *p* = 0.029). These variables can be assumed to be independent positive prognostic factors. Multivariate interaction analysis showed that solitary BM and controlled disease status significantly influenced the impact of GTR in our patient population (*p* = 0.0001).

**Table 2:** Multivariate analysis showing factors independently associated with longer OS. *P*-values of less than or equal to 0.05 are highlighted in bold

Variable	Hazard ratio	95% CI	*p*-value
Age < 65 years	0.53	0.32–0.86	**0.011**
Controlled disease status	0.66	0.47–0.94	**0.023**
Postsurgical systemic treatment	0.65	0.45–0.95	**0.029**
Solitary BM	0.40	0.23–0.69	**0.0001**

## Discussion

In this study, we analyzed outcomes in a large population of patients with surgically resected BMs and compared subgroups of patients of different ages with solitary, singular, or multiple BMs. The patients either had or did not have controlled systemic disease, and either received or did not receive postsurgical therapy. Without considering specific circumstances, we assumed that the importance of GTR for survival or recurrence control is not decisive. However, achieving GTR is highly relevant in patients with solitary BM status, controlled systemic disease, postoperative systemic treatment options, FRT, and in populations younger than 65 years. A significant survival benefit from GTR was observed in patients who did not receive targeted therapy, in contrast to those who did.

BM is a major cause of morbidity and mortality in patients with cancer; BMs also pose a significant challenge across medical specialties [21]. According to the ASCO-SNO-ASTRO guidelines, surgery may be offered to patients with BM. Patients with suspected BM who do not have a primary cancer diagnosis may benefit from surgery to confirm a diagnosis, and this also applies to patients with large tumors that cause mass effects [22]. The aim of surgery should be the complete removal of the suspicious lesion. Hence, neurosurgeons have to deliver meticulous results to avoid permanent neurological deterioration [4] and complete resection should not be attempted at all costs because surgical treatment is often associated with peri-operative complications [23, 24]. Furthermore, some authors argue that the risk of postoperative impairment should not be traded for GTR of BM [25] because no difference in OS has been found between GTR and STR [26, 27]. If a reduction in intracranial pressure and symptoms and the acquisition of tissue for molecular diagnoses can be achieved with sub-total resection, there is no justification for radical surgery at the cost of functional deterioration [25]. However, some authors have found no significant difference in the rate of postoperative complications based on the resection grade [28]. This statement is consistent with our data, which demonstrate that patients receiving GTR do not show neurological worsening more frequently than patients with STR.

This finding reinforces the hypothesis that GTR of BMs should not be entirely excluded but taken into account after contemplating specific conditions. Moreover, a trend shown by our data suggests that patients with GTR have a slightly lower rate of neurological worsening than patients with STR and that patients in the GTR group more frequently experience aphasia resolution. This assumption may be significant when determining the indication of primary surgery for BM and for second-look surgery in selected patients with residual tumors.

Many studies have demonstrated the importance of GTR [28–31]. Olesrud et al. (2019) described statistically significant longer OS in patients with no residual tumor than in patients with a measurable residual tumor [29]. In contrast to our study, the authors didn´t establish subgroups considering the metastasis status.

Yang et al. (2022) did not detect any statistical differences in OS rates or in progression when comparing patients with single versus multiple BMs; they advocated that surgery should be considered essential in appropriately selected patients, regardless of the number of BMs [32]. We analyzed our data from several points of view to test this assumption. First, the authors dichotomized the groups as patients with more than one lesion and patients with a single lesion, without considering the extracerebral status. However, this factor emerged in our analysis as a determinant of patient survival. Our Cox proportional hazards model showed that the GTR of solitary BM was associated with a significant benefit. Furthermore, patient survival was not influenced by isolated multiplicity, because we are dealing with a systemic disease. Factors such as systemic disease control and appropriate postoperative therapeutic options play a key role, as our hazard ratios show. This concept sharply reflects one of the recommendations of the ASCO-SNO-ASTRO guidelines, in which patients with multiple BMs and/or uncontrolled systemic disease are less likely to benefit from surgery unless the underlying disease is controllable through other measures [22]. In accordance with our findings, other authors have also observed that patients with single BM and controlled primary disease benefit from GTR [28, 30, 31]. As high-dose local radiotherapy leads to better control of intracerebral disease when considering complete resections alone [33] or progression and death when considering WBRT after resection or radiosurgery [34], our data show that GTR has a significant positive effect on OS and PFS when followed by FRT or WBRT, in contrast to the same protocols after STR. Moreover, the landscape of systemic therapy for primary tumors, extracranial metastatic disease as well as brain metastases has changed with the advent of targeted therapies including antibody-based therapies [35]. Our data suggest that if patients can be treated with targeted therapies, GTR loses therapeutic value in terms of survival and recurrence, while neurosurgical resection remains essential in reducing symptoms and mass effect.

Although no significantly higher surgical morbidity rate was found in patients older than 65 years [15], our analysis showed that GTR is relevant to the population below this age threshold. Other authors have also identified a KPS ≥ 70, age ≤ 65, and stable extracranial cancer as the best indications for the surgical resection of BM [36]. As indirect support for this hypothesis, other studies have noted that older age is significantly associated with the incidence of both overall and major complications and may contribute to increased postoperative morbidity [37]. On the other hand, other authors reported that the residual tumor burden strongly predicted prolonged OS, regardless of age [38].

It is important to remember that surgical outcome and prognosis depend not only on the clinical characteristics of the patient but also on the histological and molecular characteristics of the metastases [35, 39–41], preoperative preparation accuracy, surgical methodology, and intraoperative functional study using cortical mapping to avoid postoperative deficits that may reduce functional status and impair quality of life [37, 42]. GTR of BM can be achieved with lower morbidity using image-guided systems, such as preoperative functional MRI, intraoperative neuro-navigation, and use of fluorescein sodium [4, 27]. The use and dosege of corticosteroids significantly impact outcomes in patients undergoing BM resection [43], but according to the data collected in this study, this effect dissipates when considering EOR.

In summary, our findings emphasize the importance of more refined selection criteria for patients undergoing GTR for BM. Nonetheless, these results should be validated in subsequent studies.

Our study has several limitations. The first limitation is the single-center setting. Due to its importance, this topic deserves further investigation and validation with larger multicentric cohorts. Another possible limitation is that only patients eligible for surgery were included in the study. While this selection process produced a fairly homogeneous cohort, it may also introduce initial bias, meaning that the analysis only represents a portion of the entire population. Moreover, due to the high number of missing data and the insufficient size of subgroups in this single-center study, molecular characterization was not considered in this study, which may reduce the value of our results. Our group will analyze this aspect in a subsequent multicenter study. However, our aim was to resolve a dispute regarding the usefulness of targeted operation in patients with BM by clarifying the key role of neurosurgical therapy. Finally, this work does not consider supramarginal resection, a fundamental concept of neurosurgical therapy. Our group will analyze this aspect in a subsequent study.

## Conclusions

In terms of the influence on patient survival, GTR was not associated with longer OS and PFS in the entire population. However, the significant impact of GTR on patients with BM emerges as highly relevant in patients with solitary BM status, controlled systemic disease, postsurgical FRT and systemic not targeted treatment options, and in the population younger than 65 years.

## Electronic supplementary material

Below is the link to the electronic supplementary material.

Supplementary material 1: **Supplementary Table 1**: To evaluatethe overall impact of adjuvant therapy on survival, multiple Cox proportional hazards regressionmodels were constructed. Each model included age, metastatic status, and KPS as baselinecovariates and one treatment modality per model. All models were adjusted for the same baseline clinical covariates. Significant favorable associations were observed (CT, IT, TT, WBRT, FRT, or SRS). Across all models, age, metastatic status, and KPS were consistently and significantly associated with survival outcomes. Increasing age and presence of systemic metastases were associated with worse OS, while higher preoperative performance status was strongly protective. In the models including systemic therapies, immunotherapy was associated with improved survival (HR:0.76, 95% CI: 0.59–0.98, *p* = 0.037). Similarly, chemotherapy was significantly associated with better survival (HR:0.69, 95% CI: 0.57–0.83, *p* < 0.001), and targeted therapy had the strongest positive effect (HR:0.61; 95% CI: 0.48–0.78; *p* < 0.001). Among radiation modalities, FRT was significantly associated with improved survival (HR:0.78, 95% CI: 0.64–0.96; *p* = 0.017). In contrast, WBRT (HR:0.89, 95% CI: 0.75–1.07, *p* = 0.220) and SRS (HR:0.77, 95% CI: 0.53–1.12 *p* = 0.170) were not independently associated with overall survival. The table reports hazard ratios (HR), 95% confidence intervals (CI), and p-values. Values below 1 indicate a protective effect. P-values less than or equal to 0.05 are highlighted in bold. **Supplementary Table 2**: Comparison of functional outcomes by EOR. Aphasia outcomes were significantly better and aphasia worsening was significantly lowerin the GTR group, pointing to a protective effect of complete resection on language functions.KPS scores improved significantly postoperatively in both groups, with slightly greaterimprovement in the GTR group. Most neurological worsening outcomes (neurological overall, HS, VF, KPS) did not significantly differ between groups, though there was a trend favoring GTRby lower worsening rates. P-values less than or equal to 0.05 are highlighted in bold.

## Data Availability

The raw data supporting the conclusions of this article will be made available on reasonable request via the corresponding authors.
