# Supplementary material for: Complete resection of brain metastases – when does it matter?
Source: J Neurooncol. 2025 Aug 22;175(3):1299–309. doi: 10.1007/s11060-025-05193-9 (PMC12511165; doi:10.1007/s11060-025-05193-9)
Supplement: Supplementary file 1 — Supplementary material 1: Supplementary Table 1: To evaluatethe overall impact of adjuvant therapy on survival, multiple Cox proportional hazards regressionmodels were constructed. Each model included age, metastatic status, and KPS as baselinecovariates and one treatment modality per model. All models were adjusted for the same baseline clinical covariates. Significant favorable associations were observed (CT, IT, TT, WBRT, FRT, or SRS). Across all models, age, metastatic status, and KPS were consistently and significantly associated with survival outcomes. Increasing age and presence of systemic metastases were associated with worse OS, while higher preoperative performance status was strongly protective. In the models including systemic therapies, immunotherapy was associated with improved survival (HR:0.76, 95% CI: 0.59–0.98, p = 0.037). Similarly, chemotherapy was significantly associated with better survival (HR:0.69, 95% CI: 0.57–0.83, p < 0.001), and targeted therapy had the strongest positive effect (HR:0.61; 95% CI: 0.48–0.78; p < 0.001). Among radiation modalities, FRT was significantly associated with improved survival (HR:0.78, 95% CI: 0.64–0.96; p = 0.017). In contrast, WBRT (HR:0.89, 95% CI: 0.75–1.07, p = 0.220) and SRS (HR:0.77, 95% CI: 0.53–1.12 p = 0.170) were not independently associated with overall survival. The table reports hazard ratios (HR), 95% confidence intervals (CI), and p-values. Values below 1 indicate a protective effect. P-values less than or equal to 0.05 are highlighted in bold. Supplementary Table 2: Comparison of functional outcomes by EOR. Aphasia outcomes were significantly better and aphasia worsening was significantly lowerin the GTR group, pointing to a protective effect of complete resection on language functions.KPS scores improved significantly postoperatively in both groups, with slightly greaterimprovement in the GTR group. Most neurological worsening outcomes (neurological overall, HS, VF, KPS) did not significantly [file 11060_2025_5193_MOESM1_ESM.docx]

| **Treatment Variable** | **HR** | **95% CI** | **p-value** |
| --- | --- | --- | --- |
| **Immunotherapy** | 0.76 | 0.59 – 0.98 | **0.037** |
| **Chemotherapy** | 0.69 | 0.57 – 0.83 | **0.0001** |
| **Targeted therapy** | 0.61 | 0.48 – 0.78 | **0.0001** |
| **WBRT** | 0.89 | 0.75 – 1.07 | 0.220 |
| **Focal radiation (FRT)** | 0.78 | 0.64 – 0.96 | **0.017** |
| **SRS** | 0.77 | 0.53 – 1.12 | 0.170 |
| **Age** | 1.01 | 1.005 – 1.017 | **0.001** |
| **Metastasis status** | 1.36 | 1.2 – 1.54 | **0.0001** |
| **KPS** | 0.98 | 0.978 – 0.989 | **0.0001** |

**Supplementary Table 1** To evaluate the overall impact of adjuvant therapy on survival, multiple Cox proportional hazards regression models were constructed. Each model included age, metastatic status, and KPS as baseline covariates, and one treatment modality per model. All models were adjusted for the same baseline clinical covariates. Significant favorable associations were observed (CT, IT, TT, WBRT, FRT, or SRS). Across all models, age, metastatic status, and KPS were consistently and significantly associated with survival outcomes. Increasing age and presence of systemic metastases were associated with worse OS, while higher preoperative KPS was strongly protective. In the models including systemic therapies, immunotherapy was associated with improved survival (HR:0.76, 95% CI: 0.59–0.98, p=0.037). Similarly, chemotherapy was significantly associated with better survival (HR:0.69, 95% CI: 0.57–0.83, p<0.001), and targeted therapy had the strongest positive effect (HR:0.61; 95% CI: 0.48–0.78; p<0.001). Among radiation modalities, FRT was significantly associated with improved survival (HR:0.78, 95% CI: 0.64–0.96; p=0.017). In contrast, WBRT (HR:0.89, 95% CI: 0.75 – 1.07, p=0.220) and SRS (HR:0.77, 95% CI: 0.53 – 1.12 p=0.170) were not independently associated with overall survival. The table *reports hazard ratios (HR), 95% confidence intervals (CI), and p-values. P-values less than or equal to 0.05 are highlighted in bold*

| **Outcome** | **STR (%)** | **GTR (%)** | **p-value** |
| --- | --- | --- | --- |
| **HS** |  |  | 0.126 |
| - Dissipate | 20.0 | 27.6 |  |
| - Improved | 40.0 | 27.6 |  |
| - Stable | 20.0 | 38.2 |  |
| - Worsened | 20.0 | 6.6 |  |
| **Aphasia** |  |  | **0.039** |
| - Dissipate | 5.9 | 38.8 |  |
| - Improved | 41.2 | 28.6 |  |
| - Stable | 35.3 | 28.6 |  |
| - Worsened | 17.7 | 4.1 |  |
| **VF** |  |  | 0.724 |
| - Stable | 60.0 | 62.3 |  |
| - Worsened | 13.3 | 5.7 |  |
| **Neurological Worsening** | 12.6 | 7.7 | 0.115 |
| **Aphasia Worsening** | 5.5 | 1.8 | **0.037** |
| **HS Worsening** | 7.7 | 5.0 | 0.293 |
| **VF Worsening** | 3.3 | 1.6 | 0.270 |
| **Preoperative KPS (mean ± SD)** | 77.6 ± 17.9 | 79.8 ± 15.3 | 0.213 |
| **Postoperative KPS (mean ± SD)** | 81.2 ± 19.3 | 83.9 ± 17.1 | 0.179 |
| **KPS Gain (Post − Pre)** | +3.63 | +4.08 |  |
| **KPS Worsening** | 12.1 | 8.8 | 0.324 |
| **KPS (Categorical)** |  |  | 0.603 |
| - Improved | 42.7 | 48.7 |  |
| - Stable | 47.6 | 42.2 |  |
| - Worsened | 9.8 | 9.0 |  |

**Supplementary Table 2** Comparison of functional outcomes by EOR. Aphasia outcomes were significantly better and aphasia worsening was significantly lower in the GTR group, pointing to a protective effect of complete resection on language functions. KPS scores improved significantly postoperatively in both groups, with slightly greater improvement in the GTR group. Most neurological worsening outcomes (neurological overall, HS, VF, KPS) did not significantly differ between groups, though there was a trend favoring GTR by lower worsening rates. *P-values less than or equal to 0.05 are highlighted in bold*

| **Corticosteroids use** | **Timing** | **STR n(%)** | **GTR n(%)** | **HR for OS** | **p-value** |
| --- | --- | --- | --- | --- | --- |
| Yes | Preoperative | 63 (80.8%) | 321 (89.9%) | 0.87 | 0.333 |
| No | Preoperative | 15 (19.2%) | 36 (10.1%) | 0.59 | 0.134 |
| Yes | Postoperative | 81 (92.0%) | 411 (96.9%) | 0.88 | 0.296 |
| No | Postoperative | 7 (8.0%) | 13 (3.1%) | 0.66 | 0.468 |

**Supplementary Table 3** Pre- and postoperative corticosteroid use stratified by EOR, with corresponding hazard ratios (HR) for overall survival (OS) associated with GTR. Corticosteroid use was more frequent in the GTR group both pre- and postoperatively. However, no significant association between GTR and OS was observed within any corticosteroid subgroup. *P-values less than or equal to 0.05 are highlighted in bold*

| **Subgroup** | **STR – Mean OS** | **GTR – Mean OS** | **HR (95% CI)** | **p-value** |
| --- | --- | --- | --- | --- |
| Overall | 13.1 ± 20.9 | 18.4 ± 28.5 | 0.88 (0.74–1.05) | 0.162 |
| Age < 65 y | 13.2 ± 18.1 | 19.2 ± 28.7 | 0.75 (0.55–1.02) | **0.047** |
| Age ≥ 65 y | 13.7 ± 24.9 | 16.6 ± 28.2 | 0.99 (0.73–1.35) | 0.943 |
| Disease control | 12.1 ± 18.1 | 21.9 ± 28.2 | 0.68 (0.48–0.97) | **0.033** |
| No disease Control | 14.0 ± 23.3 | 15.3 ± 24.9 | 1.00 (0.72–1.38) | 0.992 |
| Postsurgical systemic therapy | 9.8 ± 19.2 | 20.7 ± 35.5 | 0.67 (0.46–0.98) | **0.038** |
| No postsurgical systemic therapy | 11.4 ± 14.5 | 19.4 ± 46.3 | 1.03 (0.72–1.48) | 0.729 |
| Solitary BM | 12.6 ± 25.4 | 34.2 ± 42.5 | 0.39 (0.22–0.68) | **0.0006** |
| Singular BM | 12.6 ± 12.3 | 19.1 ± 30.8 | 0.86 (0.61-1.23) | 0.403 |
| Multiple BM | 14.4 ± 24.8 | 11.7 ± 13.4 | 1.15 (0.80-1.65) | 0.235 |

**Supplementary Table 4** Impact of GTR on OS across clinical subgroups. The mean OS (± SD) in patients who underwent STR versus GTR, the corresponding HR and 95% CI derived from Cox proportional hazards models. Subgroup analyses include age, systemic disease control, postsurgical therapy and BM. *P-values less than or equal to 0.05 are highlighted in bold*

| **Systemic Subgroup** | **HR (GTR vs. STR)** | **95% CI** | **p-value** |
| --- | --- | --- | --- |
| **CT** | 0.70 | 0.48–1.03 | 0.070 |
| **No CT** | 0.88 | 0.64–1.19 | 0.400 |
| **ICI** | 0.64 | 0.35–1.20 | 0.167 |
| **No ICI** | 0.85 | 0.66–1.10 | 0.225 |
| **With TT** | 1.05 | 0.63–1.75 | 0.845 |
| **No TT** | 0.75 | 0.57–0.99 | **0.039** |
|  |  |  |  |
| **Radiation Subgroup** | **HR (GTR vs. STR)** | **95% CI** | **p-value** |
| **WBRT** | 0.91 | 0.64–1.30 | 0.611 |
| **No WBRT** | 0.79 | 0.57–1.09 | 0.151 |
| **FRT** | 0.64 | 0.41–0.99 | **0.044** |
| **No FRT** | 0.89 | 0.67–1.18 | 0.403 |
| **SRS** | 1.17 | 0.39–3.49 | 0.780 |
| **No SRS** | 0.83 | 0.65–1.06 | 0.145 |

**Supplementary Table 5** Distribution and survival impact of systemic therapies and radiation modalities stratified by EOR. *P-values less than or equal to 0.05 are highlighted in bold*

| **Subgroup** | **Mean PFS (± SD)** | **Cox HR (95% CI)** | **p-value** |
| --- | --- | --- | --- |
| Overall | 12.78 ± 23.80 | 0.84 (0.59–1.19) | 0.319 |
| Age ≥ 65 | 16.14 ± 30.62 | 0.53 (0.22–1.27) | 0.155 |
| Age < 65 | 14.45 ± 27.22 | 0.88 (0.54–1.44) | 0.601 |
| Postsurgical systemic treatment | 15.63 ± 31.10 | 0.72 (0.39–1.33) | 0.297 |
| No postsurgical systemic treatment | 18.28 ± 46.58 | 2.12 (0.29–15.80) | 0.462 |
| Solitary metastasis status | 26.54 ± 38.77 | 0.50 (0.21–1.23) | 0.130 |
| Systemic disease control | 16.58 ± 29.35 | 0.59 (0.37–0.96) | **0.032** |
| No systemic disease control | 10.72 ± 19.51 | 1.12 (0.67–1.86) | 0.662 |
|  |  |  |  |
| **Interaction** | **Coef. (± SE)** | **95% CI** | **p-value** |
| Age < 65 | 0.18 ± 4.95 | -9.56–9.91 | 0.971 |
| Age ≥ 65 | 1.86 ± 5.11 | -8.20–11.92 | 0.716 |
| WBRT | 7.42 ± 3.71 | 0.13–14.71 | **0.046** |
| No WBRT | 4.05 ± 3.59 | -3.02–11.11 | 0.261 |
| FRT | 2.69 ± 3.85 | -5.21–8.15 | 0.486 |
| No FRT | 1.47 ± 3.39 | -4.88–10.25 | 0.666 |
| SRS | 1.04 ± 24.05 | -46.2–48.28 | 0.966 |
| No SRS | -0.58 ± 5.94 | 12.25–11.09 | 0.923 |
| CT | 7.32 ± 3.84 | -0.22–14.87 | 0.057 |
| No CT | 3.59 ± 3.58 | -3.45–10.63 | 0.317 |
| ICI | 5.85 ± 4.06 | -2.13–13.83 | 0.150 |
| No ICI | 2.63 ± 3.01 | -3.29–8.56 | 0.383 |
| TT | 6.83 ± 4.01 | -1.04–14.71 | 0.089 |
| No TT | 4.67 ± 3.15 | -1.52–10.86 | 0.139 |
| Corticosteroids preoperative | 0.78 ± 4.96 | -8.97–10.52 | 0.876 |
| No corticosteroids  preoperative | 5.04 ± 5.77 | -6.3–16.38 | 0.383 |
| Corticosteroids postoperative | 6.38 ± 8.51 | -10.34–23.09 | 0.454 |
| No corticosteroids  postoperative | 10.43 ± 10.46 | -10.13–30.99 | 0.319 |

**Supplementary Table 6** Impact of GTR on intracranial progression. PFS (± SD), HR from univariable Cox regression models, 95% CI, and corresponding p-values for the overall cohort and clinically relevant subgroups are indicated. Interaction analyses between GTR and stratifying variables (age, radiotherapy, systemic treatment, corticosteroid use) are presented as regression coefficients (± standard error =SE) along with their 95% CI and p-values. *P-values less than or equal to 0.05 are highlighted in bold*


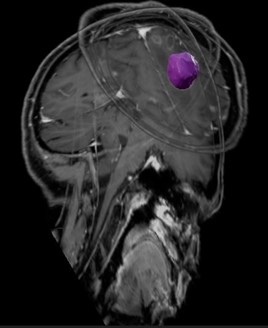


**Supplementary Figure 1** Three-dimensional representation of a brain metastasis using BrainLab software (Munich, Germany)
